# Nano-drug delivery systems in wound treatment and skin regeneration

**DOI:** 10.1186/s12951-019-0514-y

**Published:** 2019-07-10

**Authors:** Wei Wang, Kong-jun Lu, Chao-heng Yu, Qiao-ling Huang, Yong-Zhong Du

**Affiliations:** 1 Department of Pharmaceutics, Hangzhou Third Hospital, Hangzhou, 310009 China; 2 Institute of Pharmaceutics, College of Pharmaceutical Sciences, Zhejiang University, Hangzhou, 310058 China; 3 Department of Burn, Second Affiliated Hospital, School of Medicine, Zhejiang University, Hangzhou, 310009 China

**Keywords:** Nano-drug delivery system, Skin regeneration, Wound treatment

## Abstract

Skin damages are defined as one of most common lesions people suffer from, some of wounds are notoriously difficult to eradicate such as chronic wounds and deep burns. Existing wound therapies have been proved to be inadequate and far from satisfactory. The cutting-edge nanotechnology offers an unprecedented opportunity to revolutionize and invent new therapies or boost the effectiveness of current medical treatments. In particular, the nano-drug delivery systems anchor bioactive molecules to applied area, sustain the drug release and explicitly enhance the therapeutic efficacies of drugs, thus making a fine figure in field relevant to skin regeneration. This review summarized and discussed the current nano-drug delivery systems holding pivotal potential for wound healing and skin regeneration, with a special emphasis on liposomes, polymeric nanoparticles, inorganic nanoparticles, lipid nanoparticles, nanofibrous structures and nanohydrogel.

## Introduction

Skin, the largest organ of human body, functions as a pivotal barrier featured with immunologic, sensorial and protective capability. Owing to its exposure to the external environment, skin is vulnerable to a variety of external factors which result in different types of skin damage and injury. It should be noticed that the prevalence of people suffering from chronic wounds has risen sharply in recent years, due to the dramatically increasing incidence of obesity and chronic diseases such as diabetes, venous and arterial insufficiency [1]. It is estimated that chronic wounds affect about 1–2% of the European and United states population [2], for example, prevalence of diabetes ulcers alone has already reached as high as 10–22% in diabetes patients [3]. However, traditional therapies generally involve costly and long-lasting treatments with a ulcer relapse rate of above 70% [4]. The astonishing numbers of patients being eager for better healing quality and the staggering budget spent for wound care, which are still on the wax, prominently drive the research in fields of wound healing and skin regeneration.

Thanks to the innovative and impressive development of nanotechnology, numerous nano-drug delivery systems (nano-DDSs) were invented and introduced into the areas relevant to skin regeneration. It is universally testified that nano-DDSs evidently accelerate wound healing and improve the healing quality for the several prominent advantages they enjoy: (1) Nano-DDSs are found to be non-toxic, perfectly compatible with skin and favorably create a beneficial moist environment for activation and acceleration of wound healing process. (2) Some specific nano-DDSs are equipped with ability of entering into the cytoplasmic space across cellular barriers or activating specific transport mechanisms to improve the drug retention [5]. (3) When incorporated with bioactive molecules, nano-DDSs protect drugs from degradation elicited by proteases in wounds, remarkably enhancing therapeutic effectiveness [6]; (4) The sustained drug release also prolongs the maintenance of effective drug concentration, reduces the frequency of administration and leads to decline of cost as well as improvement of compliance.

This review mainly introduces the wound healing process, the current wound treatment and their limitations, and the state of the art in nano-DDSs that holds a promising potential for future application, with a special focus on liposomes, polymeric nanoparticles, lipid nanoparticles, nanofibrous structures and nanohydrogel.

## Wound categories and wound healing process

### Wound categories

Wounds are defined as the breakage or disruption of skin caused by trauma or medical/physiological conditions. Under such circumstance, damage to skin anatomical structure and the loss of skin physiological functions occur frequently. The wounds generally fall into two categories: acute wounds often result from mechanical damage or exposure to extreme heat, irradiation, electrical shock or corrosive chemicals. Such wounds heal in a relatively short period of time if supported by appropriate wound management [7]. Chronic wounds normally appear to be the complication of some specific diseases like diabetes, which is notorious for its horrendous incidence of ulcers. These wounds require for longer time to heal and their reoccurrence rates are extremely high unless the root diseases are cured [8].

According to wound depth, the wounds can be classified as three kinds: superficial wounds (only lose a part of epidermis), partial thickness wounds (epidermis and deeper dermal layers are affected) and full thickness wounds (subcutaneous fat and deeper tissue are disrupted) [9].

### Wound healing process

**Fig. 1:**
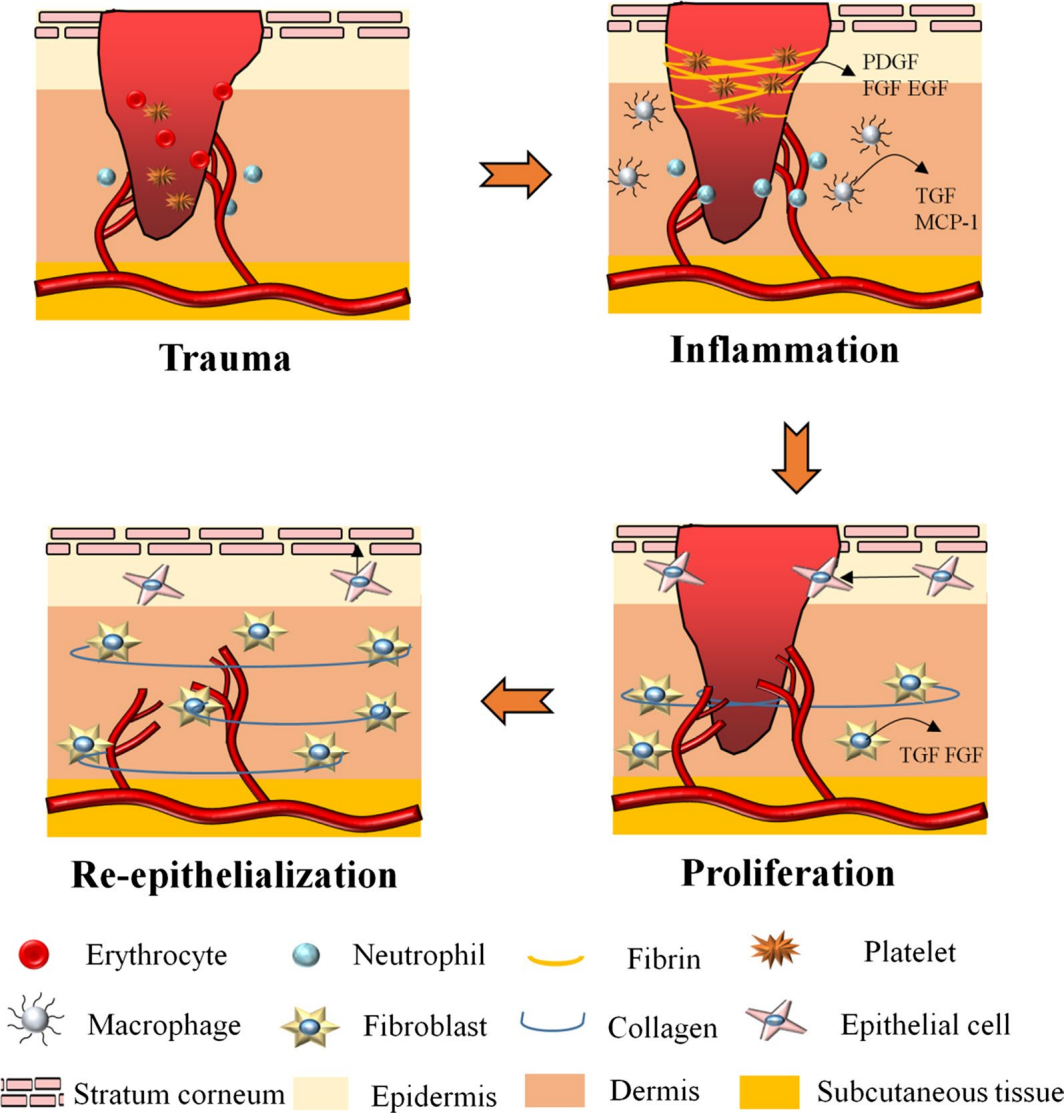
Illustration of wound healing process

Wound healing is a complex and dynamic physiological process involves with various cells, mediators, extracellular matrix (ECM) components, growth factors, and proteinases [10]. As showed in Fig. 1, it can be generally divided into three overlapping phases including inflammatory, proliferative, and re-epithelialization/remodeling phase [11, 12].

The inflammatory phase often lasts 2 to 5 days after skin damage. When an injury occurs, the hemostasis is initiated immediately by intravascular platelets to form a clot and stop bleeding [13]. Furthermore, platelets will be activated by thrombin and release several growth factors such as epidermal growth factor (EGF), insulin-like growth factor 1 (IGF-1), platelet-derived growth factor (PDGF), fibroblast growth factor (FGF), transforming growth factor (TGF-α and TGF-β) [14, 15]. These growth factors diffuse into wound tissue and serve as biological signals to attract neutrophils, monocytes, leukocytes and macrophages, which will further mediate the inflammation, protect skin from infection and secret more growth factors to accelerate wound healing [16, 17].

The proliferative phase generally takes 3 days to 2 weeks after injury, featured with cell proliferation and migration [18]. Fostered by proangiogenic factors such as PDGF released by platelets and inflammatory cells within wound area, new blood vessels and capillaries gradually take shape [19]. Simultaneously with angiogenesis, migration of fibroblasts is also elicited by the stimulation of PDGF and FGF from inflammatory cells to form granulation tissue [20, 21]. With the accumulation and proliferation of fibroblasts, new ECM composed of collagen, proteoglycans, and elastin is produced. Some fibroblasts even differentiate into myofibroblasts and play a role in the contraction of wound area [22]. Moreover, activated keratinocytes around wound margin migrate to injured area to complete re-epithelialization [23].

Re-epithelialization and remodeling phase varies from 3 weeks to 2 years post-injury. The collagen III in newly-synthesized ECM is gradually replaced by collagen I and the new born collagen fibers evolve into a more organized lattice structure, augmenting tensile strength of healed skin [24, 25]. Remodeling phase also concerns about scar formulation [26].

## Current wound treatment

The ultimate goal of the wound management is to prevent serious infection, accelerate wound healing and reduced scars and pain for patients. Currently, a set of strategies are available for wound management mainly including debridement, autografts and application of therapeutic agents. In addition, some burgeoning new therapies such as stem cell therapy, gene therapy, photothermal and photodynamic therapy, are playing an increasingly vital role in some complicated wound treatment.

### Debridement

Conventional debridement removes the necrotic or infected tissue that may prolong the inflammatory phase and impede wound contraction as well as re-epithelialization, fostering a better wound bed for healing process [27]. Debridement, including surgical, autolytic, mechanical, maggot, and enzymatic method, usually involves with further application of wound dressings [28]. Although debridement, especially the sharp debridement, has been well acknowledged as the gold standard for rapid removal of necrotic tissue and prevention of potential infection, it is still confronted with some limitations: its lasting and significant pain sometimes can be unacceptable for patients [29, 30], and it requires for experienced clinicians and specific materials in order to avoid second trauma. Therefore, applied method should be in accordance with the evaluation of wound characteristics, patient condition, and resources available in treatment [31].

### Autografts and allografts

The use of autografts and allografts remains the gold standard for skin regeneration. Autografts and allografts approaches mainly harvest full-thickness fascia from a donor site of patients or other donators and graft it over the target region [32]. Autografts have reputation for excellent adhesion to the wound site and better cosmetic results, conspicuously relieving pain and reducing rejection. However, the rigorous requirement of donor site limits their usage and such skin grafts also bring the undesirable amount of scar and serious skin contraction in late stage of wound healing, along with the costly hospital stay [33]. As for allografts, the major advantage is the temporary prevention of wound dehydration and contamination, along with the favorable fitness to wound. Nevertheless, due to the resource of grafts, the risk of disease transmission and higher rate of immune rejection are inevitable [34]. It should be noticed that the tissue-engineered skin substitutes and in situ biofabrication of skin substitutes like cultured epithelial autografts, are emerging as promising strategies to overcome the setbacks of traditional autografts [35].

### Topical drug application

Application of topical drugs, which mainly focuses on promoting healing process and preventing infection, still plays an indispensable role in treatment for all types of wounds. Hence, a large demand still exists in exploring novel therapeutic agents for topical wound therapy. Topical therapeutic agents consist of growth factors and antimicrobial agents being crucial for the wound treatment and skin regeneration. Growth factors are biologically active polypeptides which regulate cell growth, differentiation, and migration and exert an impact on all stage of wound healing. It has been confirmed in some clinical researches, growth factors exert amazing effects on wound healing promotion and skin function restoration without any obvious side effects. The clinical used growth factors are listed in Table 1.

**Table 1:** Growth factors in clinical application

Growth factor	Target cells	Administration	Function	Refs.
EGF	Fibroblasts Keratinocytes	Topical	Promote cell proliferation, differentiation and migration; accelerate epidermal regeneration	[93]
PDGF	Neutrophils Macrophages Fibroblasts Smooth muscle cells	Topical	Increase the structural integrity of vessels; promote cell proliferation, ECM Deposition and re-epithelialization	[94]
bFGF	Keratinocytes Fibroblasts	Topical	Promote collagenase production, ECM deposition and re-epithelialization	[95]
GM-CSF	Keratinocyte Endothelial cells Macrophages Eosinophils	Topical/subcutaneous injection	Promote local recruitment of inflammatory cells, stimulate cell proliferation and differentiation and wound contraction	[96]
TGF-β	Keratinocytes Macrophages Lymphocytes Fibroblasts	Topical	Promote granulation tissue formation; re-epithelialization; matrix formation and remodeling	[97]

Owing to infection being a leading cause of mortality and horribly delaying the wound healing, antimicrobial agents are generally administrated both topically and systemically. The choice of antimicrobial agents strongly depends on the microbiological analysis for species and susceptibility of microorganisms. The most commonly used antimicrobial agents are listed in Table 2.

**Table 2:** Most commonly used antimicrobial agents in wound treatment

Antimicrobial agents	Administration	Spectrum	References
Gentamicin	Systemic/topical	Gram-positive bacteria	[98]
Tetracycline	Oral/topical	Gram-positive and Gram-negative bacteria	[99, 100]
Ciprofloxacin	Oral/systemic	Gram-positive and Gram-negative bacteria especially Gram-negative bacilli	[101, 102]
Vancomycin	Systemic	Gram-positive bacteria especially MRSA	[103]
Penicillin G	Systemic	Non-β-lactamase-producing Gram-positive bacteria, anaerobes	[104]
Neomycin	Systemic/topical	Aerobic Gram-negative bacilli and Gram-positive aerobes	[105]
Polymyxin B	Systemic	Gram-negative bacteria	[106]
Mupirocin	Topical	Gram-positive bacteria especially MRSA, some Gram-negative flora	[107]
Amphotericin B	Systemic/topical	Fungi	[108]
Silver sulfadiazine	Topical	Gram-positive, most Gram-negative bacteria, and some fungal forms	[109]
Mafenide acetate	Topical	Gram-negative bacilli, anaerobes	[110]

## Nano-drug delivery system in wound treatment and skin regeneration

**Fig. 2:**
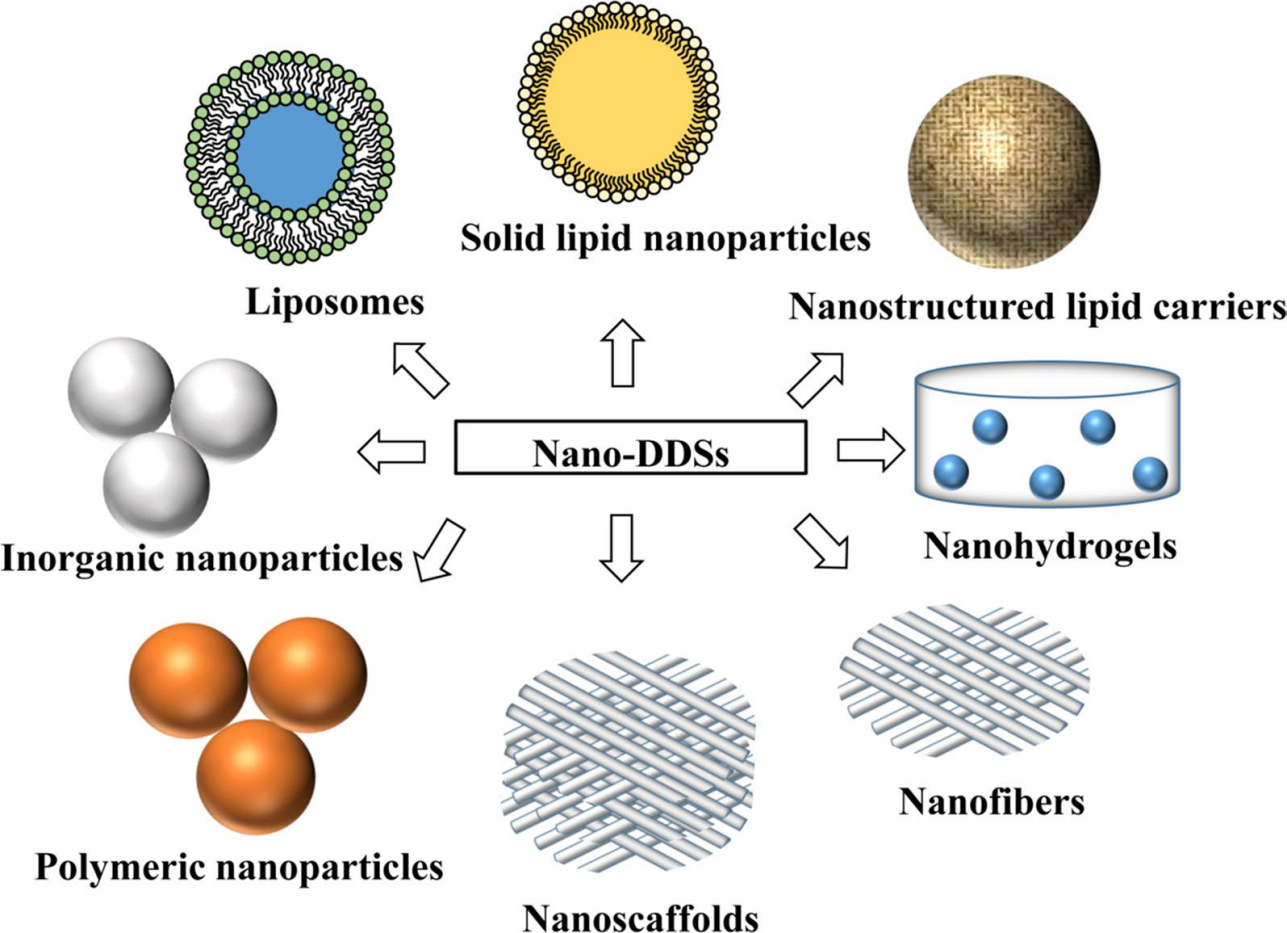
Nano-drug delivery systems in skin regeneration and wound treatment

Nano-DDSs hold immense potential in enhancement of drug therapeutic efficacy for their capability of preventing drug degradation and sustaining drug release. Numerous nano-DDSs carrying therapeutic agents are springing up unprecedentedly and adopted in promoting wound healing and skin regeneration, mainly including liposomes, polymeric nanoparticles, inorganic nanoparticles, lipid nanoparticles, nanofibrous structures and nanohydrogel [36–38] (Fig. 2). Recent researches of nano-DDSs are listed in Table 3.

**Table 3:** Recent research of nano-drug delivery system in wound treatment and skin regeneration

Formulation	Drug	Administration	Outcome	Refs.
Liposomes	bFGF	Smearing wound every 3 days	Accelerated the wound closure of mice with deep second-degree scald, expedited regeneration of vascular vessels	[43]
Liposomes	Madecassoside	Smearing wound once a day for 12 days	Enhanced permeation and distribution in skin so as to exhibit superior burn wound healing effect	[111]
Liposomal membrane	Usnic acid	Topical treatment applied every 4 days	Enhanced maturation of granulation tissue and better collagen deposition	[44]
Deformable liposomes	EGF, PDGF-A and IGF-1	Topical treatment, once a day for 11 days	Significantly enhanced the healing of chronic wounds due to synergistic effect of complex	[47]
Deformable liposomes	Curcumin	Topical treatment, once a day for 18 days	Shorten inflammatory process, prevent infection and promoted fibrosis, angiogenesis, re-epithelialization and wound contraction	[49]
Deformable liposomes	Baicalin	Daily topical treatment for 3 days	Complete skin restoration and inhibition of inflammatory markers such as oedema, TNF-α and IL-1β	[48]
Nanoparticles	Thrombin	Topical treatment	Advanced process of healing, improved skin tensile strength, reduced complications in surgery	[66]
Nanoparticles	Silver	Topically given with dressing every day	Rapid healing and improved cosmetic appearance were achieved via reduction in wound inflammation, and modulation of fibrogenic cytokines	[64]
Nanoparticles	Cerium oxide	Topical treatment once a day for 13 days	Reduced the oxidative stress at wound site and protected regenerative tissue, providing favorable environment for restoration	[112]
Nanoparticles	ZnO_2_	Topical treatment	Had good antibacterial activity and accelerated wound healing in animal model	[65]
Hydrogel loading nanoparticles	Asiatic acid/ZnO/CuO	Topical treatment	Raised DNA, total protein, hexosamine and hydroxyproline content and rendered superior re-epithelization, collagen fibers arrangement and angiogenesis	[67]
Hydrogel loading nanoparticles	Silver oxide	Local injection	Showed excellent antimicrobial activity and burn wound healing in the second burn rat model	[113]
Nanoparticles	LL37	Intradermally injection	Significantly promoted granulation, collagen deposition, re-epithelialized and neovascularized composition	[59]
Microspheres/scaffold	Mupirocin	Applied topically, covered and tied	Combated infection and stimulated fibroblast proliferation and dermal collagenization	[114]
Nanoparticles	Norfloxacin	Applied topically	Sustained drug release to 24 h, retained antimicrobial efficacy and exhibited good stability	[60]
Nanoparticles	Amphotericin B	Applied topically every other day	Equipped with strong anti-fungi capacity and quicker efficiency in fungal clearance	[61]
Nanoparticles	hVEGF gene/stem cells	Intramuscularly injection	Facilitated angiogenesis and limb salvage, also reduced muscle degeneration and tissue fibrosis	[62]
SLNs/NLCs	EGF	Administered topically twice a week	Significantly improved wound closure, restoration of the inflammatory phase, and re-epithelialization	[72]
SLNs	LL37 and A1	Applied topically	Promoted wound closure in fibroblast cells and keratinocytes, simultaneously enhanced antibacterial activity	[73]
NLCs/scaffold	Andrographolide	Applied every other day 21 days	Enhanced the wound healing with no scar and improved tissue quality	[74]
Bioadhesive gel containing SLNs	Cyclosporine A	Administered topically twice a day	Significantly increased rate of mucosal repair	[115]
Nanofibers	Lawsone	Locally delivered every three days over a period of 14 days	Significantly increased in TGF-β1 and Collagen gene expression in vitro and promoted re-epithelialization of the wound in vivo	[80]
Nanofibrous dressing	Astragaloside IV	Locally delivered at the wound site every 2 days	Stimulated wound closure, increased angiogenesis, regulated newly formed types of collagen, and collagen organization	[81]
Nanofibrous membranes	Collagen/Zein	Applied topically/single	Exerted antibacterial activity and induced fast tissue regeneration	[116]
Nanofibers loaded with nanoparticles	Cefazolin/zinc oxide	Applied topically/single	Showed great anti-bacterial activity, enhanced cell adhesion and epithelial migration, contributed to faster and more efficient collagen synthesis	[117]
Nanofibrous scaffold	Human bone marrow stem cells	Applied topically	Boosted cell growth rate and accelerated wound recovery	[118]
Scaffold loaded with nanoparticles	Silver	Applied topically	Strong anti-microbial capability and excellent biocompatibility with fibroblast cells	[83]
Nanofibrous scaffold carrying with nanoparticles	PDGF-BB and VEGF	Topically placed on the wound site	Accelerated tissue regeneration and remodeling, promoted angiogenesis	[119]
Hydrosol/scaffold	TiO_2_	Applied topically	Strongly inhibited the growth of Staphylococcus aureus and induced red blood cells aggregation to stop bleeding	[84]
Nanofibrous scaffold carrying with nanoparticles	Fe_3_O_4_	Applied topically	A suitable scaffold for cell adhesion with favorable magnetic behavior and low cytotoxicity	[85]
Hybrid nanostructures/hydrogel	Ag/Ag–AgCl/ZnO	Applied every 2 days for 14 days	Stimulated the immune function, produced the synergistic antibacterial effects and accelerated wound healing	[120]
Nanohydrogel	Nanosilicates/VEGF	Applied topically	Enhanced cell adhesion and spreading, reduced blood clotting time, facilitated in vitro tissue regeneration and wound healing	[92]
Nanohydrogel	K_2_(SL)6K_2_	Subcutaneous injection	Provoked an inflammatory response, stimulated host cells to secret wide range of cytokines, so as to promote cell recruitment and angiogenesis	[121]
Nanohydrogel	Acrylic acid	Applied topically	Maintained the activity and morphology of human dermal fibroblasts, promoted rapid cell proliferation, and affected 9 gene expression related to wound healing	[91]
Nanohydrogel	Baicalin	Topically smeared every day for 4 days	Faster and more complete skin restoration and inhibition of specific inflammatory markers were noticed	[90]
Nanohydrogel	Ultrashort aliphatic peptides	Applied topically	Earlier onset and completion of autolytic debridement as well as faster wound closure compared with a commercial product	[122]

### Liposomes

Liposomes are bilayer vesicles built by amphiphilic molecules such as phospholipids, emerging as one of promising nano-carriers for topical drug delivery [39]. They are nontoxic, biodegradable, biocompatible with skin, and capable of accommodating both hydrophilic drugs (e.g. growth factors) in inner water cavity and hydrophobic agent in bilayer [40, 41]. In this way, liposomes provide protection for encapsulated drug and sustain the drug release. Furthermore, liposomes effectively cover wound and create moist environment on wound surface after application, which is very conducive to wound healing [42]. Taking advantage of all these merits, liposomes have been universally applied in wound treatment and skin regeneration. Xu et al. [43] prepared a novel liposome with hydrogel core of silk fibroin which effectively encapsulated bFGF. The vehicles remarkably improved the stability of bFGF in wound fluids and maintained cell proliferation activity with respect to traditional liposomes. Furthermore, the liposomes with hydrogel core efficiently accelerated wound healing, particularly in inducing angiogenesis. Nunes et al. [44] evaluated the promoting effect of a gelatin-based membrane containing usnic acid-loaded liposome on wound healing. The experiments on a porcine model indicated that the liposomal membrane conspicuously controlled the secondary infection. In addition, more exuberant and cellularized granulation tissue with better collagen deposition was observed in the liposomal membrane treated group, therefore the special membrane boasted a comparable capacity to commercial product DuoDerme with regard to enhance maturation of granulation tissue and scar repair.

Presented as a new generation of liposomes, deformable liposomes, also called transfersomes, mainly consist of phospholipids and an edge activator (such as sodium cholate, sodium deoxycholate and Tween-80) [45], bringing new insight into topical drug delivery. These novel carriers not only integrate the benefits of traditional liposomes, but show more merits in topical application. The presence of edge activator renders high flexibility of deformable liposomes and enables them to across the stratum corneum and reach the viable epidermis [46]. Uk Choi et al. [47] conjugated low-molecular-weight protamine (LMWP) to the N-termini of EGF, PDGF-A and IGF-1, these molecules were subsequently complexed with hyaluronic acid and eventually incorporated into cationic deformable liposomes. The results showed that the cationic elastic liposomes containing the growth factor complex significantly accelerated the wound closure rate in the diabetic mouse model, with the maximal shrink of wound size by 58% compared with the native growth factor complex. It was fully confirmed that the elastic liposomes cooperated with growth factor complex, synergistically exerting both rapid and prolonged effects on promoting chronic wound healing. A new self-assembling core–shell gellan-transfersome loading baicalin was designed by Manconi et al. [48], they found out that novel transfersomes showed a relatively high skin drug deposition, about 11% of baicalin was retained in the whole skin, 8% of which was in the dermis, considered to be quite efficient. Daily application of baicalin transfersomes in mice model brought about complete skin restoration and inhibition of inflammatory markers such as oedema, TNF-α and IL-1β. Kianvash et al. [49] also noticed that their newly prepared propylene glycol nanoliposomes containing curcumin not only featured by preferential physiochemical properties (small size, sustained drug release, good stability and biocompatibility), but promoted second degree burns in rat model in terms of avoiding infections and elevating wound contraction.

Nevertheless, liposomes also exhibit some demerits in application: drug leakage in liposomes sometimes can be unavoidable and rapid [50, 51]; the low reproducibility and stability of liposomes remains a major obstacle for its expansion in clinical use [52, 53].

### Polymeric nanoparticles

Polymeric nanoparticles are biocompatible colloidal systems drawing increasing attention in both biomedicine and bioengineering fields [54]. When embedded or conjugated with these polymeric devices, drugs are protected from degradation by the proteases presenting in the wound and released in a controlled manner so as to reduce administration frequency. The need of effectively delivering biomolecules such as antimicrobial agents, growth factors and genes, will be met with aid of nanoparticles [55, 56]. Currently most polymeric nanoparticles are prepared by poly lactic-co-glycolic acid (PLGA, crowned as the mostly used polymers), alginate, gelatine, chitosan, as well as other polymer combinations [57, 58].

Many researches focus on developing polymeric nanoparticles encapsulating antimicrobial agents. Chereddy et al. [59] reported a PLGA nanoparticle loaded with antimicrobial peptide LL37 (PLGA-LL37 NPs) could be a biodegradable drug delivery system that accelerated healing process. It displayed antimicrobial activity on *Escherichia coli* and induced promoted cell migration while lifting no effect on proliferation of keratinocytes. In full thickness excisional wound model, PLGA-LL37 NP-treated group exhibited advanced granulation tissue formation, characterized by significant higher collagen deposition, re-epithelialized composition and neovascularization. Furthermore, it improved angiogenesis and modulated the inflammatory wound response by up-regulation of interleukin-6 (IL-6) and vascular endothelial growth factor (VEGF). Dave et al. [60] prepared a lipid-polymer hybrid nanoparticle formulation which was able to sustained drug release to 24 h with favorable skin permeation and reduced the frequency of application. Norfloxacin -loaded nanoparticles still performed well in antimicrobial efficacy test against *Staphylococcus aureus* and *Pseudomonas aeruginosa*. Therefore, it was considered to hold a broad prospect in treating burn-induced infections.

To reduce high cytotoxicity of Amphotericin B and improve the patient appliance, Sanchez et al. [61] incorporated Amphotericin B into a silane-based hydrogel nanoparticles to replace the traditional intravenous injection infusion. Amphotericin B nanoparticles resulted in equivalent or enhanced killing efficacy with 72.4–91.1% by 4 h for Clinical strains. It also contributed to statistically significant reduction of fungal biofilm metabolic activity ranging from 80 to 95%. In a murine full-thickness burn model, Amphotericin B nanoparticles cleared fungi in a more rapid manner versus free drug solution within 3 days while their wound healing rates were similar.

Performance of nanoparticles also exceeds expectation in gene therapy related to skin regeneration. To overcome the setbacks of insufficient expression of angiogenic factors and low cell viability after transplantation, biodegradable nanoparticles were developed to deliver hVEGF gene to human mesenchymal stem cells and human embryonic stem cell-derived cells [62]. hVEGF production, cell viability, and engraftment into target tissues of stem cells were prominently enhanced. The scaffold seeded with genetically modified stem cells directly achieved 2–4 fold higher vessel densities 2 weeks post-implantation versus control cells or cells transfected with hVEGF gene by Lipofectamine 2000. When intramuscularly injected into mouse ischemic hindlimbs, cells pretreated with nanoparticles still facilitated angiogenesis and limb salvage, meanwhile, reducing muscle degeneration and tissue fibrosis.

### Inorganic nanoparticles

Inorganic nanoparticles refer to nanoparticles deprived from inorganic materials, including the metallic nanoparticles, carbon based nanoparticles, ceramic nanoparticles etc. [63]. Benefiting from its intrinsic nature of materials, inorganic nanoparticles exhibit both the similar merits in wound healing treatment and strong antibacterial effect, for example, silver nanoparticles are often applied as antimicrobial agents. Therefore, the combination of inorganic nanoparticles is more preferred in research to achieve synergistic promoting effect of both materials and drugs.

Jun et al. [64] investigated the promoting wound-healing effect of silver nanoparticles and its mechanism were systematically revealed in burn wound and chronic wound model. Dose-dependent rapid healing and improved superficial wound appearance were observed and further studies demonstrated that the relatively prompt wound healing and reduced wound inflammation may be mediated by elevation of TGF-β, VEGF, IL-6 induced by silver nanoparticles.

Ali et al. [65] designed and synthesized ZnO_2_ nanoparticles by co-precipitation method. This kind of nanoparticles is an efficient inorganic material with antibacterial properties. ZnO_2_ nanoparticles had good antibacterial activity for *pseudomonas aeruginosa* and *aspergillus* isolated from wound infected tissues of burn patients. The results of histopathological evaluation confirmed that ZnO_2_ nanoparticles could accelerate the healing of skin wounds in animal models in vivo.

Thrombin is a widely used drug for topical hemostasis and wound healing. With the aim of overcoming the weak stability of drug, a thrombin-bounded maghemite (Fe_2_O_3_) nanoparticle were fabricated and its therapeutic effect were evaluated via application on an incisional wound model [66]. The results illustrated that the nanoparticle-treated group characterized by fewest inflammatory cells, smallest amount of granulation tissue along the surgical scar and highest values of skin tensile strength. All the evidences supported that thrombin-bounded maghemite nanoparticle remarkably advanced the wound healing stage and achieved better healing quality.

Studies have been extended to the blending application of different inorganic nanoparticles to render a better efficacy. Thanusha et al. [67] developed a hydrogel co-encapsulated with asiatic acid and nanoparticles (zinc oxide and copper oxide) for second burn wound healing. Physicochemical studies showed the formulation was characterized by porous morphology, large water uptake, excellent tensile strength and good anti-bacterial capacity. Thanks to the co-loaded nanoparticles, in vivo study of therapeutic efficiency demonstrated that DNA, total protein, hexosamine and hydroxyproline content of wounds in hydrogel treated group were all raised significantly; re-epithelialization, collagen fibers arrangement and angiogenesis were confirmed to be more superior than the group treated with commercial available products.

### Lipid nanoparticles

Solid lipid nanoparticles (SLNs) and nanostructured lipid carriers (NLCs) were representatives of lipid nanoparticles introduced to overcome the limitation of liposomes. Lipid nanoparticles were generally prepared with physiological lipids or lipid molecules and their preparation process requires no involvement of any potentially toxic organic solvents [68]. Their nontoxic colloidal dimensions contribute to the controlled release of drug and versatility of administration [69, 70]. The potential of lipid nanoparticles for topical therapeutic or cosmetic purposes has been partially exploited as a market product loaded with Q10 is available (NanoRepair Q10^®^, Dr. Rimpler) [71].

Gainza et al. [72] reported both SLNs and NLCs loading with rh-EGF for treatment of chronic wounds. Both of the nano-formulations were prepared through emulsification-ultrasonication method, but the preparation process of NLCs involved no organic solvent and characterized by much higher encapsulation efficiency. The results of the effectiveness of nano-formulations showed that both of them equipped with superior capability on promoting cell proliferation compared with free rh-EGF, and significantly improved healing in terms of wound closure, restoration of the inflammatory process, and re-epithelialization when applied on full-thickness wound model in db/db mice.

Fumakia et al. [73] fabricated SLNs loading with an elastase inhibitor serpin A1 and antimicrobial peptide LL37 to achieve synergistic effect on wound healing. Making the most of the synergistic effect of drugs and extraordinary characteristics of SLNs, the formulation promoted wound closure in fibroblast cells and keratinocytes. In addition, it simultaneously enhanced antibacterial activity against *S. aureus* and *E. coli* compared with the group treated with LL37 or A1 alone.

In another related study, andrographolide-loaded lipid nanoparticles were developed, optimized, then successfully incorporated into a chitosan-HA scaffold [74]. This scaffold exhibited appropriate porosity, suitable swelling ratio and a controlled drug release behavior up to 72 h. When applied to second degree burn wounds, it notably reduced scar formation and improved healing quality, which could be explained by anti-inflammatory and antioxidant effect of chitosan, HA and nanoparticles. Therefore, this scaffold would be a potential candidate for wound healing treatment.

### Nanofibrous structures

Nanofibers are fabricated from natural and synthetic continuous polymer chains which are able to subsequently act as nanofibrous sheets or 3D-scaffolds applied in tissue engineering [75, 76]. These nanofibrous structures are designed to mimic the ECM, provide favorable condition for cell attachment and elevate cell-drug interaction, serving as a replacement for artificial dermal analogues [76, 77]. Electrospinning is the most widely-adopted technique for production of nanofibers. An electrical charge is taken as driving force to draw fibers from a polymer solution so as to fabricate nanometric continuous fibers [78]. Due to its high area to volume ratio, the nanofibers enhance transfer of a variety of therapeutic agents including diverse antimicrobial agents, growth factors and even nucleic acids [79].

Adeli-Sardou et al. [80] electrospun lawsone into polycaprolactone-gelatin (PCL/Gel) polymers in core–shell architecture, so as to produce special nanofibers for skin tissue regeneration. In addition to boosted cell attachment and proliferation brought along by nanofibers, results of RT-qPCR revealed that in vitro gene expression of TGF-β1, collagen and EGF was impressively elevated in the nanofiber-treated cells. Furthermore, 1% lawsone PCL/Gel had the best impact on wound healing of rats, especially in the acceleration of re-epithelialization. Shan et al. [81] prepared astragaloside IV loaded silk fibroin/gelatin nanofibrous dressing via electrospinning nanotechnology. The nanofibrous dressing was equipped with excellent biocompatibility, significantly improving cell adhesion and proliferation in vitro, accelerating wound healing and inhibiting scar formation in vivo. Results also found out that the nanofibrous dressing exerted positive impacts on angiogenesis, collagen production and collagen organization.

Combined with stem cell therapy, Ramasatyaveni et al. [82] attached mouse bone marrow stem cells to a porous polyethylene glycol-polyurethane (PEG-PU) scaffold to better fulfill differentiation potential and wound healing capability of stem cells. The results of in vivo observations depicted significant increase in fibroblast proliferation, collagen deposition and anti-oxidant enzyme activity, with obvious decreased expression of pro-inflammatory cytokines (IL-1β, TNF-α, IL-8, etc.) and concomitant increase in anti-inflammatory cytokines (IL-10, IL-13) at an early healing stage. Furthermore, enhanced engraftment and vascularity were detected to provide evidences for an accelerated wound tissue closure.

In some cases, nanofibrous structures were simultaneously integrated with other nano-formulations to achieve a synergic impressive effect on skin regeneration. Zulkifli et al. [83] fabricated the hydroxyethyl cellulose-silver nanoparticles into scaffold to endow it with anti-microbial capability. Fibroblast cells were able to adhere onto the surface of scaffolds after co-incubation, indicating that it is a potential substrate with high biocompatibility for biomedical applications, especially in the wound healing and tissue engineering field. According to report of Fan et al. [84], a nanofibrous scaffold carrying with nano-TiO_2_ hydrosol was designed for better skin repair. The results of physicochemical properties revealed the good permeability and stability, which offer a humid environment for wound healing and met the requirement of wound coverage protection. Due to the embedment of nano-TiO_2_, the scaffold strongly inhibited the growth of *Staphylococcus aureus* and induced red blood cell aggregation to stop bleeding. In another study, magnetic iron oxide nanoparticles were incorporated into three-dimensional fibrous scaffolds to form a novel formulation and its physicochemical properties and cell biocompatibility in vitro were investigated [85]. It turned out that magnetic iron oxide nanoparticles were successfully loaded into scaffolds while maintaining magnetic behavior. It was also a suitable scaffold for cell adhesion with low cytotoxicity, thus having prominent advantages in skin tissue engineering, particularly in the treatment that may encounter magnetic field.

### Nanohydrogel

Nanohydrogel is the three-dimensional polymeric networks considered as ideal formulation for wound management: the porous three-dimensional structure endows it with the ability of aqueous fluid absorption [86], preventing wound dehydration and creating a beneficial moist environment for wound healing [87]; its non-adhesive nature allows it to preserve the wound bed while maintaining the penetration of oxygen, which is necessary for wound healing [88]; soft texture of nanohydrogel provides comfortable experience in the course of treatment [89]. Furthermore, nanohydrogel is able to encapsulate many related drugs with perfect compatibility and efficacy, exerting an impressive effect on skin regeneration.

A gellan-cholesterol nanohydrogel embedding baicalin was introduced to speed up wound healing process [90]. Characterized by proper viscosity, improved skin retention and preferable biocompatibility, it was further applied to a cutaneous inflammation mice model induced by a phorbol ester. The baicalin-loaded nanohydrogel exhibited optimal performance for a complete skin restoration and inhibition of specific inflammatory markers (i.e., myeloperoxidase, tumor necrosis factor-α and oedema) were realized in vivo. Xi Loh et al. [91] found that a newly-produced bacterial nanocrystal cellulose/acrylic acid hydrogel could rapidly adhere to fibroblasts, maintain the activity and morphology of human dermal fibroblasts, limit cell migration, promote rapid cell proliferation, and affect 9 gene expression related to wound healing like IL-6, IL-10, GM-CSF, TGF-β and matrix metalloproteinase-2 (MMP-2). Thus this hydrogel was regarded as playing a pivotal role in skin regeneration.

Besides, the versatile administration of nanohydrogel has received considerable interest, with the special focus on injectable nanohydrogel. Giriraj et al. [92] reported a nanocomposite hydrogel consisted of natural polysaccharide, κ-carrageenan and nanosilicates. This specially designed nanohydrogel was confirmed to feature with high mechanical stiffness and good porosity with an interconnected network. With the addition of VEGF, VEGF-loaded nanohydrogel significantly enhanced cell adhesion and spreading, reduced blood clotting time and facilitated in vitro tissue regeneration. However, the further investigation in vivo is required to fully reveal the therapeutic efficacy of this novel formulation on wound healing.

## Conclusion

The treatment of chronic wounds or ulcers remains a thorny and daunting challenge because current therapies mostly failed to provide favorable outcomes of wound healing. Nevertheless, the progressive expansion of nano-DDSs in recent years has brought new insight for skin regeneration of wounds: these drug carriers prolong drug release, protect drug from degradation and improve skin retention, so as to realize augment of the therapeutic power of biological and synthetic molecules (e.g. reduction or eradication of the wound bacterial load and improvement of re-epithelialization). Moreover, various combinations of nano-DDSs are served as synergistic platforms for delivery, some of which even mimic and offer perfect physiological environment for the healing process. Despite the enormous potential of nano-DDSs, these systems also have exposed some limitations in researches such as lack of international standards and evaluation methods on their toxicology, biocompatibility and targeting efficiency, as well as the undeniable restriction of industrial production for their complicated preparation procedures. However, it is an inevitable and unstoppable trend for researchers to further exploit the full potential of nano-DDSs, overcome the technical difficulties and bring tangible benefits for the patients suffered from wounds, nano-DDSs are bound to constitute the most promising and cost-effective therapies to boost the wound healing and skin regeneration.

## Data Availability

Not applicable.

## Abbreviations

nano-DDSs: nano-drug delivery systems
ECM: extracellular matrix
EGF: epidermal growth factor
IGF-1: insulin-like growth factor 1
PDGF: platelet-derived growth factor
FGF: fibroblast growth factor
TGF: transforming growth factor
PLGA: poly lactic-co-glycolic acid
IL-6: interleukin-6
VEGF: vascular endothelial growth factor
SLNs: solid lipid nanoparticles
NLCs: nanostructured lipid carriers
GM-CSF: granulocyte-macrophage colony-stimulating factor
MMP: matrix metalloproteinase
